# Hyper IgE in Childhood Eczema and Risk of Asthma in Chinese Children

**DOI:** 10.3390/molecules21060753

**Published:** 2016-06-10

**Authors:** Chantel Ng, Kam Lun Hon, Jeng Sum Charmaine Kung, Nga Hin Pong, Ting-Fan Leung, Chun Kwok Wong

**Affiliations:** 1 Department of Pediatrics, Prince of Wales Hospital, The Chinese University of Hong Kong, Hong Kong, China; chantel_ng@yahoo.com (C.N.); jsckung@gmail.com (J.S.C.K.); b688755@mailserv.cuhk.edu.hk (N.H.P.); tfleung@cuhk.edu.hk (T.-F.L.); 2 Institute of Chinese Medicine, The Chinese University of Hong Kong, Hong Kong, China; ck-wong@cuhk.edu.hk; 3 Department of Chemical Pathology, Prince of Wales Hospital, The Chinese University of Hong Kong, Hong Kong, China

**Keywords:** atopic dermatitis, atopy, eczema, prognosis, NESS, severity

## Abstract

*Background:* Atopic eczema is a common childhood disease associated with high IgE and eosinophilia. We characterized the clinical features associated with hyper-IgE (defined as IgE > 2000 IU/L) in eczema. *Methods*: Nottingham Eczema Severity Score (NESS), family and personal history of atopy, skin prick test (SPT) for common food and aeroallergens, highest serum IgE ever and eosinophil counts were evaluated in 330 children eczema patients. Childhood-NESS (NESS performed at <10 years of age) and adolescent-NESS (NESS performed at >10 years of age) were further analyzed. *Results*: IgE correlated with NESS (spearman coefficient 0.35, *p* < 0.001) and eosinophil percentage (spearman coefficient 0.56, *p* = 0.001). Compared with IgE ≤ 2000IU/L (*n* = 167), patients with hyper-IgE (*n* = 163) were associated with male gender (*p* = 0.002); paternal atopy (*p* = 0.026); personal history of atopic rhinitis (*p* = 0.016); asthma (*p* < 0.001); dietary avoidance (*p* < 0.001); use of wet wrap (*p* < 0.001); traditional Chinese medicine use (TCM, *p* < 0.001); immunomodulant use (azathioprine or cyclosporine, *p* < 0.001); skin prick sensitization by dust mites (*p* < 0.001), cats (*p* = 0.012), dogs (*p* = 0.018), food (*p* = 0.002); eosinophilia (*p* < 0.001); more severe disease during childhood (*p* < 0.0001) and during adolescence (*p* < 0.0001), but not onset age of eczema or maternal atopy. Logistic regression showed that hyper-IgE was associated with personal history of asthma (exp(B) = 5.12, *p* = 0.002) and eczema severity during childhood and adolescence (*p* < 0.001). For patients <10 years of age, dust mite sensitization (*p* = 0.008) was associated with hyper-IgE. For patients >10years of age, food allergen sensitization was associated with hyper-IgE (*p* = 0.008). *Conclusions:* Hyper-IgE is independently associated with asthma, more severe atopy and more severe eczema during childhood and adolescence. IgE > 2000 IU/L may be a tool to aid prognostication of this chronic relapsing dermatologic disease and its progression to asthma.

## 1. Introduction

Childhood eczema is a chronic distressing disease [1,2,3,4,5,6,7]. Disease onset is usually before five years of age in the majority of patients [2,6,8]. The prevalence of atopic diseases among these individuals and their family members is high and includes eczema, asthma or allergic rhinitis. Atopy is also evidenced in laboratory tests (such as positive skin prick reaction to common food and aeroallergens or elevated serum IgE levels above laboratory reference range for age) [4,6,7,9,10]. Very high levels of IgE are encountered in many of these patients. Hyper-IgE or Job’s syndrome is a distinct condition associated with STAT3 mutation, severe primary immunodeficiency, increased serum IgE levels (>2000 IU/L), recurrent infections and atopic dermatitis (AD)-like skin lesions [11,12,13,14,15]. Hyper-IgE syndrome and atopic eczema with elevated IgE are two different diseases with eczematous skin lesions. However, patients with atopic eczema have impaired barrier skin function and allergic sensitization which are absent in patients with hyper-IgE syndrome. Furthermore, there are other phenotypes of patients having both manifestations of eczema and a raised IgE, namely Immunodysregulation polyendocrinopathy enteropathy X-linked (IPEX) syndrome, DOCK 8 deficiency, and phosphoglucomutase 3 deficiency. We reviewed clinical and atopic features of patients with childhood atopic eczema and evaluated the clinical significance of hyper-IgE (>2000 IU/L) in these patients.

## 2. Methods

The clinical data of Chinese patients with atopic eczema (AE) followed consecutively at the pediatric dermatology clinic of a university hospital were reviewed. Eczema was diagnosed according to the U.K. Working Party’s Diagnostic Criteria for Atopic Dermatitis [16]. Disease severity was assessed by the Nottingham Eczema Severity Score (NESS). NESS is a self-administered questionnaire with a validated Chinese version which evaluated a patient’s eczema severity over the preceding 12 months [17,18,19]. NESS is routinely recorded in the first visit, and was randomly scored in subsequent visits. NESS further categorized AE severity into three groups: mild, moderate or severe. Family and personal history of atopy, use of wet wrapping, dietary avoidance, traditional Chinese medicine, immunomodulants (azathioprine or cyclosporine), skin prick test for common foods (egg, milk, soybean, peanut, mixed nuts, fish, shellfish, beef, pork, orange, tomato) and aeroallergens (house dust mite *D. pteronysissnus* and *D. farina*, cat, dog, cockroach, grass pollen), highest serum total IgE ever (and age of measurement), and highest eosinophil counts ever were analyzed. Hyper-IgE is defined as IgE above 2000 IU/L. In case of missing data, hard copy, electronic medical records and the investigator’s own database were accessed. In a subset of patients whose first visits were prior to 10 years of age, childhood-NESS (NESS performed at <10 years of age) were recorded. Adolescent-NESS (NESS performed at age 10 years) were also recorded if patients presented after 10 years of age or were followed up beyond 10 years of age. Fisher’s Exact test for categorical variables were performed. For continuous variables, Mann-Whitney U tests and Krustal-Wallis one-way ANOVA were used. Logistic regression were analyzed using SPSS Statistics 20 (IBM, New York, NY, USA). Probability values less than 0.05 were considered significant. This is a review of clinical data of patients followed at our outpatient clinic over the years. The ethics application was obtained from the University Ethics committee for this review.

## 3. Results

The IgE levels and medical records of 330 patients were available for this review. IgE correlated with NESS (spearman coefficient 0.35, *p* = 0.001 (two-tailed)) and eosinophil percentage (spearman coefficient 0.56, *p* < 0.001). Compared with IgE ≤ 2000 IU/L (*n* = 167), patients with hyper-IgE (*n* = 163) were associated with male gender (*p* = 0.002); paternal atopy (*p* = 0.026); personal history of atopic rhinitis (*p* = 0.016); asthma (*p* < 0.001); dietary avoidance (*p* < 0.001); use of wet wrapping (*p* < 0.001); traditional Chinese medicine use (TCM, *p* < 0.001); immunomodulant use (azathioprine or cyclosporine, *p* < 0.001); skin prick sensitization by dust mites (*p* < 0.001), cats (*p* = 0.012), dogs (*p* = 0.018), food (*p* = 0.002); eosinophilia (*p* < 0.001); more severe disease during childhood (*p* < 0.0001) and during adolescence (*p* < 0.0001), but not onset age of eczema or maternal atopy (Table 1, Table 2 and Table 3).

Spearman correlation showed IgE also correlated with disease severity (NESS measured during childhood: 0.411, *p* < 0.001; NESS during adolescence: 0.393, *p* < 0.001). Using skin prick dust mite sensitization, food allergen sensitization, personal history of allergic rhinitis, personal history of asthma, and the NESS, logistic regression showed that hyper-IgE was associated with a personal history of asthma (exp(B) = 5.12, *p* = 0.002) and eczema severity during childhood and adolescence (*p* < 0.001). For patients < 10 years of age (*n* = 122), dust mite sensitization (*p* = 0.008) was associated with hyper-IgE. For patients > 10 years of age (*n* = 270), food allergen sensitization was associated with hyper-IgE (*p* = 0.008). IgE correlated with NESS (spearman coefficient 0.35, *p* < 0.001 (two-tailed)) and eosinophil percentage (spearman coefficient 0.56, *p* < 0.001).

## 4. Discussion

Childhood eczema is a common chronic relapsing disease associated with atopy and elevated IgE [1,2,6,20]. Allergic rhinitis and asthma were significantly more prevalent in patients with AE (odds ratio for AR was 2.9 (CI: 1.3–6.5) and for asthma was 4.3 (CI: 1.3–16.10)) [6]. Prognostically, parents are very concerned to know if their children with eczema will improve (or outgrow it) as they grow older, and if they will go on to develop asthma and airway allergies (atopic march) [6].

*Hyper-IgE and eczema severity.* Serum IgE levels have been shown to correlate with the severity of atopic eczema [10,21]. In this study, IgE correlated with NESS (spearman coefficient 0.35, *p* = 0.001). In a prospective correlational study of 117 patients, we demonstrated that the IgE level correlates significantly with various objective clinical scores (including Scoring Atopic Dermatitis (SCORAD)) and chemokine markers of AD [10]. Furthermore, higher IgE levels were seen in subjects with atopic dermatitis with no improvement in skin eruptions, whereas those with improvement had low IgE levels [22]. Total serum IgE has also been shown to increase with age [23], but Sampson and colleagues reported that patients with persistent disease are not as severely affected as they were in infancy [24]. However, the strength of association between IgE and atopic eczema varies between studies. In the present cohort of 330 patients with atopic eczema, hyper-IgE (IgE > 2000 IU/L) is significantly correlated with the severity of eczema in childhood and adolescence. This suggests a role for IgE > 2000 IU/L as a prognostic implicator for eczema severity and intractability. Hyper-IgE is a factor, among other clinical features of atopy, that may alert clinicians to a more severe and prolonged course when they manage their young eczema patients.

New studies proposed a novel concept of atopic eczema/dermatitis syndrome (AEDs) that is classified into being intrinsic and extrinsic. The extrinsic form of AEDs is the IgE-mediated form and it is associated with respiratory allergies and high serum IgE levels [25]. This classification remains a topic of controversy, with higher serum levels of IgE being the only subjective way to differentiate between the two [26]. The precise cutoff value for IgE has not been determined, however. Our study showed that those with hyper-IgE had significantly increased eczema severity during childhood and adolescence which matched the observation that milder disease severity is seen in those with non-IgE-mediated–type AEDs. More research has to be done to evaluate whether this could serve as a simple cutoff value for the classification of AEDs.

Over the years, many studies have tried to look into the associating factors of eczema severity and disease persistence. Roth and colleagues reported 50 years ago that AE persisted into adult life in approximately 70% of patients with severe AE and in 60% of those with milder disease. [27] A family history of atopy was found in 66% [27]. A personal history of asthma, hay fever, urticaria, migraine headaches and rhinitis was found in 55%. They also demonstrated that the determination of the eosinophil count in the blood was not a useful tool in predicting the severity or course of AE [27]. Rystedt *et al.* demonstrated that the prognostically unfavorable factors influencing eczema were, in order of importance, severe (widespread) dermatitis in childhood, family history of AE, associated allergic rhinitis and/or bronchial asthma (with allergic rhinitis as the dominant of these two factors), female sex and early age at onset. [28]. Wuthrich *et al.* followed 121 children with infantile eczema and found persistence into adult life occurring in 63%, with 32% having a chronic continuous course. Prognostically unfavorable factors were early-onset severe disease within the first six months of life and very high serum IgE levels [29]. Our previous study of 383 adolescent patients conferred with Garmhausen and colleagues that severe disease in adolescence was associated with AE onset in infancy, aeroallergen sensitization, high IgE level, eosinophil count, but not with family or personal atopy status [30,31].

*Hyper-IgE and asthma.* The theory of “Atopic March” favors the consideration of eczema as a systemic disease, and indicates that many children with AE go on to develop asthma and allergic rhinitis as their eczema improves with time [2,5,6,32]. Serum IgE levels are increased in atopic phenotypes such as those with asthma and allergic rhinitis. This association is controversial with atopic eczema, particularly with the debate on non-IgE-mediated AEDs. Wakamori and colleagues analyzed IgE levels in children without atopic disorders but dry skin alone and showed that the level of IgE was significantly raised compared to healthy subjects. They observed that these patients with xerosis alone developed atopic dermatitis and allergic rhinitis in later years [22]. Our patients with hyper-IgE had significantly more allergic rhinitis and asthma. The exact role of IgE antibody sensitization in the disease process has yet to be clarified. Although there is uncertainty in whether IgE sensitization may influence overall eczema prognosis, hyper-IgE seems to be a factor that predicts patients that will progress along the path of “atopic march” to develop asthma. Furthermore, our personal unpublished data seem to suggest that IgE status may be concordant with time; namely, patients with hyper-IgE tend to remain hyper-IgE. It remains to be demonstrated if the measurement of one IgE, among other clinical factors, may be useful in prognosticating childhood asthma and eczema severity into adolescence and adulthood.

We used NESS instead of other common scores for the determination of disease severity and eczema chronicity over a 12-month interval [17,18,19]. Severity scores such as SCORAD or SASSAD are less appropriate because they merely assess disease severity over a one- to two-week period during which eczema severity may wax and wane dramatically with weather changes, psychosocial issues and superimposed *S. aureus* infection [33,34,35]. Neither SCORAD nor EASI would have been a more adequate outcome measure because both assess the acute symptomatology of eczema. Nevertheless, we previously demonstrated that IgE correlates significantly with objective SCORAD and chemokine markers of AD, and is a useful indicator for predicting moderate to severe AD in children [10].

Documentation of disease severity (NESS) before and after 10 years of age was not available in some of the patients, precluding the ability of studying the subjects in a case control manner. Also, IgE levels were not checked at the same time point in the disease in all patients because many were managed elsewhere before referral to our tertiary centre. Ideally disease severity and IgE should be assessed at the time of onset and serially to document progress during toddlerhood, childhood, adolescence and adulthood in a large prospective cohort. Furthermore, in a tertiary hospital setting, the population of patients encountered and followed is likely to be biased towards more severe disease [20]. Hence, our conclusions may not be generalizable.

In conclusion, hyper-IgE in patients with eczema is independently associated with asthma, more severe atopy and more severe eczema during early childhood and adolescence. IgE > 2000 IU/L may be a tool to aid prognostication of this chronic relapsing dermatological disease and its progression to other atopic disorders.

## Figures and Tables

**Table 1:** Subject characteristics (data in median; IQR unless stated).

Subject Characteristics	Non-Hyper (%) (*n* = 167)	Hyper-IgE (%) (*n* = 163)	*p* Value
Sex (m %)	83 (49.7)	109 (66.9)	0.002 *
Age of IgE measured in years	11.6; 8.6	13.4; 6.0	0.000 *
IgE (IU/mL)	547; 792	6440; 11496	0.000 *
Age of NESS < 10 year (*n* = 122)	6.3; 3.7	6.9; 2.3	0.100
NESS Score (mean + SD)	10.9 ± 3.2	13.0 ± 2.4	0.000 *
Mild AD	16 (23.9)	3 (5.5)	-
Moderate AD	14 (20.9)	8 (14.5)	-
Severe AD	37 (55.2)	44 (80)	-
Age of NESS > 10 year (*n* = 270)	15.2; 4.2	15.5; 4.0	0.373
NESS Score (mean + SD)	9.1 ± 3.4	11.5 ± 2.5	0.000 *
Mild AD	48 (37.5)	15 (10.6)	-
Moderate AD	44 (34.4)	44 (31.0)	-
Severe AD	36 (28.1)	83 (58.5)	-

* *p*-value smaller than 0.05 is considered statistically significant.

**Table 2:** Hyper-IgE and clinical parameters.

Clinical Parameters	Non-Hyper (%) (*n* = 167)	Hyper-IgE (%) (*n* = 163)	*p* Value
Onset age ≤ 1 year	86 (51.5)	91 (56.2)	0.439
Paternal atopy	62 (37.1)	81 (49.7)	0.026 *
Maternal atopy	57 (34.1)	56 (34.4)	0.966
Personal AR	114 (68.3)	131 (80.4)	0.016 *
Personal asthma	56 (33.5)	89 (54.6)	0.000 *
Highest Eosinophil	7.9% ± 5.5%	13.7% ± 7.0%	0.000 *
SPT (>4 mm)			
Dust mites (*n* = 305)	124 (84.4)	153 (96.8)	0.000 *
Cat (*n* = 305)	43 (29.3)	69 (43.7)	0.012 *
Dog (*n* = 304)	19 (12.9)	37 (23.6)	0.018 *
Food allergens (*n* = 305)	94 (63.9)	127 (80.4)	0.002 *

* *p*-value smaller than 0.05 is considered statistically significant.

**Table 3:** Hyper-IgE and treatment.

Treatment	Non-Hyper (%) (*n* = 167)	Hyper-IgE (%) (*n* = 163)	*p* Value
Food avoidance ever	109 (65.3)	136 (83.4)	<0.0001 *
Wet wrap ever	25 (15.0)	102 (62.9)	<0.0001 *
TCM ever	87 (52.1)	116 (71.2)	<0.0001 *
Immunomodulant ever	2 (1.2)	35 (21.5)	<0.0001 *

TCM: Traditional Chinese medicine; immunomodulant: azathioprine or cyclosporine; * Fisher’s exact test is performed.

## Abbreviations

The following abbreviations are used in this manuscript:

AE	Atopic eczema
AR	Allergic Rhinitis
CDLQI	Children Dermatology Quality of Life
NESS	Nottingham Eczema Severity
SCORAD	Scoring Atopic Dermatitis
SPT	Skin prick test
TCM	Traditional Chinese Medicine
